# Poisoning by Lead-shot Left in a Bottle

**Published:** 1846-11

**Authors:** 


*Poisoning by Lead-shot left in a Bottle.*—An individual was seized with violent colic and symptoms of poisoning, after having drunk several glasses of liquor. Dr. Hohl, who was called to the patient, examined the liquid, and found it to be turbid instead of clear; and on pouring it out into a glass, he discovered at the bottom of the bottle two pellets of shot which had become firmly fixed and converted by corrosion to carbonate of lead. On examining them, he found only a very small nucleus of metallic lead in the centre of each: so long as the liquor was clear, no ill effects had followed its use,—these had only occurred when the turbid portion, near the bottom of the bottle, had been taken. The liquor was proved to hold suspended a salt of lead, from which the symptoms of poisoning had undoubtedly arisen. This shows that great care should be used in cleansing bottles; and that a few shot left in them may give rise to all the symptoms of lead-poisoning.—*Lond. Med. Gaz.*

